# Limited day-to-day variation in the canine gut microbiota: implications for microbiome studies

**DOI:** 10.3389/fvets.2025.1632686

**Published:** 2025-07-28

**Authors:** David Atuahene, Luke Wolfe, Oona Elisabet Vanhatalo, Benjamin T. Veenstra, Joseph H. Skarlupka, Katie L. Anderson, Garret Suen, Giorgia Meineri, Jessica C. Pritchard

**Affiliations:** ^1^Department of Veterinary Sciences, School of Agriculture and Veterinary Medicine, University of Turin, Turin, Italy; ^2^Department of Bacteriology, University of Wisconsin-Madison, Madison, WI, United States; ^3^Department of Medical Sciences, University of Wisconsin-Madison School of Veterinary Medicine, Madison, WI, United States

**Keywords:** day-to-day variation, dogs gut microbiome, fecal sampling, microbiome assessment, non-invasive sampling

## Abstract

The gut microbiome is vital for health and affects gastrointestinal, systemic, and neurological functions. In dogs, fecal samples provide an effective mechanism to assess the gut microbiota as they are non-invasive, easily obtained, and representative of the gut microbiome. However, traditional methods usually require sampling across three consecutive days per time point to minimize the presumed variation in the gut microbiome. Here, we sought to investigate whether the gut microbiome obtained from a single-day fecal sampling is reflective of the microbiome obtained from three-day collections. To accomplish this, we collected fecal samples from 12 dogs over 3 days and compared each single-day microbiome against the combined microbiotas of the three-day samples. We found no significant daily variation in the gut microbiota, as determined by two one-sided tests of equivalence (TOST) analysis, indicating that there are little to no day-to-day changes in the microbiota. Further microbial comparisons using PERMANOVA (*p* = 0.98) and non-metric multidimensional scaling also showed no significant differences in the microbial composition across the sampled days. Taken together, our findings suggest that a single sample can represent the gut microbiome as accurately as samples obtained across three consecutive days. As such, a single-day sampling approach can be used in dog microbiome studies, which would reduce both labor and costs while preserving overall data quality.

## Introduction

The gut microbiome plays a vital role in maintaining health and, more recently, has been in the spotlight because of its influence on gastrointestinal function and broader systemic and neurological mechanisms. Fecal samples are widely utilized as a proxy for assessing the gut microbiome in dogs and have become an accepted sampling method, as feces provides a non-invasive proxy for the microbial community residing in the gastrointestinal tract (1).

The potential fluctuation in the gut microbiota poses a considerable challenge to studies that rely on single or multi-point sampling. Therefore, in order to guarantee that stool samples offer a reliable representation of the gut microbiome, current methods often use a three-day sampling period to reduce inter-sample variability and hence, account for potential transient microbial fluctuations (2, 3). However, these multi-day protocols are both labor-intensive and costly, which presents a barrier that can limit the scalability of studies, particularly when aiming to explore large populations or the effects of long-term treatments.

In this study, we sought to address whether single-day fecal sampling could represent the microbiome similarly to a three-day sampling regimen. If validated, this approach could significantly streamline the process, reducing labor and financial burdens while maintaining the robustness of microbiome assessments (4, 5). This would provide further insights into the stability of the gut microbiome and, by extension, how microbial data should be interpreted concerning gut microbiome outcomes in dogs.

## Materials and methods

### Study population and sample collection

This study included 12 client-owned dogs with epileptic history as part of a larger clinical study focused on antiepileptic therapy and gut microbiota composition. All dogs were older than 1 year of age, received daily stable antiepileptic medications, and had not been administered antibiotics 6 months prior to the commencement of the study. All dog owners provided their informed consent (IACUC no. V006528-R01) at the time of enrollment. All subjects remained on a stable commercial diet for the full 90 days of study. While individual diets varied among dogs, each dog was required to consistently consume the same diet throughout the study period, with no added probiotics or dietary changes permitted. Occasional treats were allowed, but owners were instructed to avoid treats containing probiotics or dairy products. Investigators conducted regular check-ins every 6 weeks to ensure each subject followed their diet, took their medication, and monitored their overall health throughout the study. Fecal samples were collected from each dog over three consecutive days (days 88, 89, and 90), yielding 36 total samples from the 12 subjects. The dog owners collected fecal samples, froze at −4°C to −20°C, and then transported to the laboratory on ice after the 3-day collection period. Upon receipt, the samples were stored at-80°C until processing.

Detailed sample information regarding the dogs used in this study can be found in Supplementary Table 1.

### DNA extraction

DNA extraction by a mechanical cellular disruption method and treatment with phenol:chloroform was conducted using the method described by Henderson et al. (18). Briefly, approximately 150 mg of each fecal sample was added to a 2 mL bead-beating tube containing 500 μL of 2 × sodium chloride-tris-EDTA (STE) buffer (Sigma-Aldrich, USA, Cat. No. S7899) and 300 mg of 1.0 mm diameter zirconia/silica beads (BioSpec Products, USA, Cat. No. 11079110z). The samples were homogenized by vortexing and centrifuged at 500 × *g* for 15 min at 4°C. An additional 800 μL of 2 × STE buffer was added, and up to 1,000 μL of the supernatant was transferred to a new bead-beating tube containing 0.1 mm diameter zirconia/silica beads and a 4 mm stainless steel bead (Qiagen, Germany, Cat. No. 69965).

For chemical lysis, 115 μL of an enzymatic cocktail (comprising 50 μL lysozyme (10 mg/mL) (Sigma-Aldrich, USA, Cat. No. L6876), 10 μL mutanolysin (1 mg/mL) (Sigma-Aldrich, USA, Cat. No. M9901), 5 μL lysostaphin (5 mg/mL) (Sigma-Aldrich, USA, Cat. No. L7386), and 50 μL 20% sodium dodecyl sulfate (Sigma-Aldrich, USA, Cat. No. L3771)) was added to each sample, followed by 700 μL phenol:chloroform:isoamyl alcohol (25:24:1) (Sigma-Aldrich, USA, Cat. No. P2069). The tubes were vortexed, incubated at 56°C for 30 min, and then bead-beaten for 3 min using a Mini-BeadBeater-24 (Biospec Products, USA, Cat. No. 112011). After centrifugation at 16,000 × *g* for 10 min at 4°C, the top aqueous layer was transferred to a new 2 mL tube and subjected to additional washes with 500 μL phenol: chloroform: isoamyl alcohol (25:24:1) until a clear and colorless aqueous layer was obtained.

The aqueous layer was precipitated with 70 μL of 3 M sodium acetate (Sigma-Aldrich, USA, Cat. No. S7899) and 700 μL of isopropanol. The samples were incubated at-20°C for 30 min to 1 h. DNA was pelleted by centrifugation at 16,000 × *g* for 20 min at 4°C, washed twice with 70% ethanol (Sigma-Aldrich, USA, Cat. No. E7023), and dried using a Savant SpeedVac (Thermo Scientific, USA, Cat. No. SPD1030DDA). The dried DNA pellets were resuspended in 100 μL of TE buffer (Invitrogen, USA, Cat. No. AM9849), stored at 4°C overnight, or dissolved at 37°C for 1 h. Finally, DNA was purified using a NucleoSpin Gel and PCR clean-up kit (Macherey-Nagel, Germany, Cat. No. 740609) and quantified using the PicoGreen assay (Thermo Fisher Scientific, USA, Cat. No. P11496) in a microplate reader (BioTek Instruments, USA, Cat. No. FLx800). All extracted DNA samples were stored at −80°C for long-term preservation.

### Library preparation

Universal bacterial primers (F-GTGCCAGCMGCCGCGGTAA; R-GGACTACHVGGGTWTC TAAT) with adapters for Illumina-based sequencing and unique barcodes were used to amplify the V4 region of the 16S rRNA gene as described by Kozich et al. (19). The PCR reactions contained 25–50 ng of DNA, 0.2 μmol/L of each primer, and 2 × KAPA HiFi HotStart ReadyMix (KAPA Biosystems, Wilmington, MA, USA) in a final volume of 25 μL. The PCR cycling conditions were as follows: Initial denaturation at 95°C for 3 min, followed by 25 cycles of 95°C for 30 s, 55°C for 30 s, and 72°C for 30 s, with a final extension at 72°C for 5 min. After PCR, the products were separated on a 1% (w/v) low-melt agarose gel stained with SYBRSafe DNA gel stain (Invitrogen, Waltham, CA, USA). Bands of approximately 380 bp indicated successful amplification and were purified using a Zymoclean Gel DNA Recovery Kit (Zymo Research, Irvine, CA, USA). Negative controls without template DNA were included in each PCR run. If a band appeared in the negative control, the entire set of samples was re-amplified. If no band was observed in the negative control, the area corresponding to the expected amplicon size (~380 bp) was excised and sequenced to verify the absence of contamination. After purification, the DNA was re-quantified using a Qubit fluorometer (Invitrogen, Waltham, MA, USA), and an equimolar library was constructed from all PCR products at a final concentration of 4 nmol/L.

### 16S rRNA sequencing and microbiome analysis

The library was sequenced on an Illumina MiSeq using an Illumina MiSeq v2 2 × 250 bp paired-end sequencing kit, and the resulting data were processed using the mothur pipeline (v1.48.1) (6). Initial quality control of the sequence reads was performed using the screen.seqs function with the following parameters: maxambig = 0, minlength = 200, maxlength = 500, and maxhomop = 8. Sequence reads were first screened and aligned to our region of interest. Then, the aligned sequences were further filtered for our region of interest using the “screen.seqs” function with the following parameters: start = 1,968, end = 11,550. The sequences were filtered to identify unique reads. Similar sequence reads were grouped together using “pre.cluster” with the parameter diff = 2. Chimeras were identified and removed using “chimera.uchime” and “remove.seqs” functions, respectively. The sequence reads were then classified using the SILVA taxonomy database (Release 138) (7) and any reads mapped to non-bacterial organisms (including chloroplast, mitochondria, archaea, eukaryotes, and unknown taxonomies) were removed using the “remove.lineage” function. “Split.abund” was also used to remove sequence reads that appeared only once in the entire dataset. Finally, “cluster.split” was used to collapse sequences into operational taxonomic units (OTUs) with the parameters method = opti, cutoff = 0.03.”Unique sequences were grouped using the unique.seqs function and aligned to the SILVA 16S rRNA gene reference alignment database (Release 138). The sequences generated in this study were submitted to the National Center for Biotechnology Information’s Sequence Read Archive under BioProject Accession PRJNA1233570.

### Statistical analysis

All statistical analyses were performed using the R statistical program (v4.4.1) as previously described by Skarlupka et al. (8). Briefly, sequencing coverage was assessed using Good’s coverage (9), and samples with less than 95% coverage were excluded. Three datasets were generated to determine whether using samples from a single day versus combining data from all 3 days produces equivalent results. The first dataset comprised counts from a randomly selected day for each dog (the “random dataset”). Additionally, the mean and median counts across the 3 days for each dog were included in the second and third datasets, referred to as the “mean dataset” and “median datasets,” respectively. All three datasets were subjected to natural log transformation to ensure the normality of the sample counts.

We then conducted two one-sided tests of equivalence (TOST): one between the “random dataset” and the “mean dataset” and the other between the “random dataset” and the “median dataset.” Both standard and robust TOSTs were performed with the “equivalence” package in R Studio using the “test” function, with epsilon set at 0.25. This equivalence margin was selected based on precedent in similar microbial ecology studies where temporal stability was evaluated (8). Of note, the robust TOST is a nonparametric test that does not assume normality and is highly robust against outliers and long-tailed distributions (8).

To assess daily changes in the overall microbial composition for each dog, we examined the beta diversity of the samples. Initially, OTU count tables from all days were rarefied to match the read depth of the sample with the lowest number of reads. Rare taxa, which include all OTUs with less than 0.1% abundance across all samples, were removed from our analysis. Subsequently, we conducted a nonmetric multidimensional scaling (NMDS) analysis of the filtered rarefied table using the Bray–Curtis dissimilarity matrix, employing the “metaMDS” function from the “vegan” R package (10). with the parameter distance = “bray.” Finally, a PERMANOVA test with 10,000 permutations was conducted on the dissimilarity matrix to assess differences across days with dog ID considered as a block.

## Results

### Microbial sequencing and OTU analysis

From the 36 samples collected in our study, initial sequencing yielded 1,483,169 raw sequence reads; after filtering, a total of 1,074,962 sequence reads remained. Of these, 32 samples met a sufficient coverage threshold greater than 0.95, as assessed using Good’s index. The remaining four samples (two samples from dog 3: time points 1 and 2; one sample from dog 6: time point 3; and one sample from dog 12: time point 3) had an average Good’s index of 0.6 (SD = 0.15), and these samples were deemed to have low sequence coverage. As such, all samples from these dogs were removed from our analysis (Supplementary Table 2). The only exceptions were for the TOST analysis, where only the low-coverage samples were removed (i.e., all other viable time points from each of the 3 dogs were retained) and the mean TOST analysis where only the single sample from dog 3 was excluded.

The number of sequences per sample ranged from 8,113 to 68,659, with an average of 33,587 ± 20,447 and a median of 33,018 sequences. These sequences were clustered into 964 unique operational taxonomic units (OTUs) with 97% sequence similarity. To estimate richness, we calculated the observed OTUs, and the number of OTUs within our dataset varied from 48 to 347 per sample, with a mean of 140.4 ± 74.75 and a median of 130.

### TOST analysis and day-to-day microbiota stability

**Figure 1:**
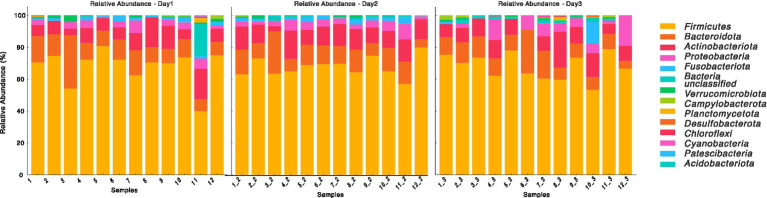
Relative abundance of bacterial phylum over time (Days 1, 2, and 3). The stacked bar plots represent the relative abundance of various bacterial taxa at the phylum level across different samples (labeled 1 to 12) on Day 1, Day 2, and Day 3. The taxa are color-coded and displayed in the legend on the right and Relative abundance is expressed as a percentage on the y-axis.

To assess the day-to-day variation in the microbiota of these dogs, we applied the TOST analysis to all animals within the same OTU table. The TOST tests comparing OTU abundances from a random day sample with the mean or median was found to be significant (*p* < 2e−16), and this result did not differ. This supports the one-sided alternative hypothesis of statistically equivalent day-to-day variation in the gut microbiota and further indicates no variation over successive days. Furthermore, we assessed the relative abundance of phyla across the samples on the 3 days using a two-way ANOVA with Phylum as Factor 1 and Day as Factor 2. Day had no significant effect (*F*(1,308) ≈ 0, *p* = 1.00), and the interaction between Phylum and Day was also not significant (*F*(13,308) = 0.35, *p* = 0.98). These results confirm that the relative abundance of phyla did not exhibit statistically significant changes across the 3 days (Figure 1).

### Rarefaction and NMDS analysis

**Figure 2:**
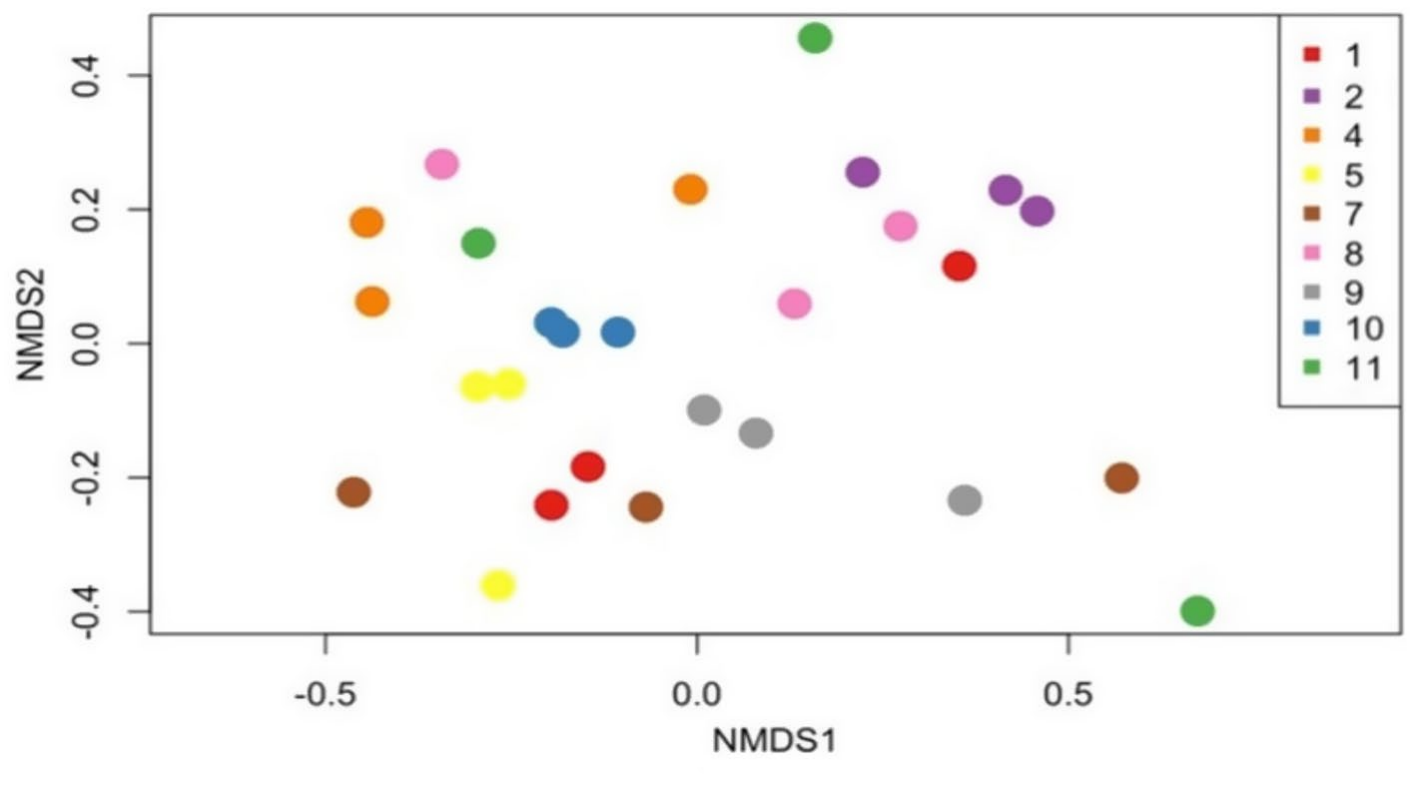
Day-to-day gut microbiota composition analysis. The non-metric multidimensional scaling (NMDS) plot illustrates Bray–Curtis dissimilarity indices, depicting the gut microbiota composition over three consecutive days in seven dogs. Each point on the plot represents the microbial composition of a sample, with colors denoting individual dogs (as shown in the legend). The PERMANOVA results (*p* = 0.98) indicate no significant day-to-day differences in the microbiota composition.

We then randomly subsampled sequences by performing a rarefaction analysis with a minimum total read count of 8,113. This removed 386 OTUs from our initial analysis (Supplementary Table 3). Three of the 12 stool samples were excluded from these analysis as they did not meet the Good’s index threshold. The Bray–Curtis dissimilarity index was then used to compare samples, considering all samples on the same day as one group from the nine dogs across the 3 days. When considering day-to-day variation, a PERMANOVA analysis showed a non-significant result (*p* = 0.98), suggesting that there were no significant differences in microbiota composition across the 3 days. Non-metric Multidimensional Scaling (NMDS) also did not reveal any distinct separation in microbiota composition (Figure 2).

Code for mothur pipeline is available at https://github.com/datuahene/DaytoDayVariationProject.

## Discussion

Here, we investigated the day-to-day variability of the dog gut microbiota using fecal samples. Due to their monogastric digestive system, fecal samples are commonly used as a proxy for their gut microbiome, thereby making fecal matter a good representative of the microbial communities in the gut (11). This method has enabled numerous studies exploring how the gut microbiome influences canine health and the potential effects of different treatments (12, 13).

In practice, three consecutive days of sampling are often used to represent the gut microbiota as a mechanism to reduce any potential variation in the gut microbiome on a day-to-day basis (12, 14). However, this approach requires higher labor investment in addition to an added cost in sample preparation and sequencing. Our study shows that a single-day fecal sample is representative of the gut microbiota collected over three consecutive days. Importantly, our statistical analyses, including TOST, PERMANOVA, and NMDS, support the relative stability of the gut microbiota in dogs over the short term (3 consecutive days). We used OTU clustering approach for consistency with previous canine microbiota studies and compatibility with our mothur-based pipeline. OTUs remain a standard in many microbiome studies, especially when comparing microbial community structure.

As reflected in our results, we found no significant day-to-day changes in the gut microbial community of dogs, with one fecal sample being sufficient to represent the gut microbial community. Given that the fecal microbiome is a good proxy for the overall gut microbiome, both in healthy and non-healthy animals (5), our data supports the usage of a single-day sampling to adequately capture the gut microbiome in dogs; however, given the absence of a healthy control group, these findings may not be generalized to broader canine populations without further study. We note that although the animals in our study had a history of epilepsy, they were otherwise healthy and had not experienced any epileptic episodes during our study. As such, we posit that our findings likely translate to both healthy and non-healthy canines, although this remains to be tested.

We also note that our study is also in concordance with previous studies demonstrating low intra-individual variation in the canine gut microbiota over short to moderate timeframes. For instance, Garcia-Mazcorro et al. (12) observed stable microbial profiles in healthy dogs over several weeks, and Barko et al. (15) further reinforced the notion of microbiome resilience in the absence of major perturbations. Moreover, Martínez-López et al. (16) found that the composition of the gut microbiota of dogs under the same feeding regime remained consistent across 18 weeks in a 6-week, 3-period crossover feeding experiment, which further confirms that the gut microbiome in dogs is relatively stable.

These results have significant implications for the design of future studies. Single-day sampling can enable more extensive study designs, reduce associated costs, and lighten the burden on both the subjects and the researchers. Moreover, it simplifies the logistics of sample collection and allows for larger sample sizes or more frequent sampling within the same resource constraints (15).

However, it is important to acknowledge some limitations of our work. We note that our sample size was comparably small, with 11 out of 12 enlisted dogs reaching the threshold value of Good’s index for analysis and 12 dogs for the relative abundance phyla estimation. However, considering the nature of this study and the challenges associated with acquiring client-owned pets for inclusion in such research, we consider the sample size practically reasonable and representative within the constraints of real world veterinary clinical studies. Moreover, our study does not consider other factors that influence the gut microbiota, such as environmental changes and genetic background (17). This study’s findings are limited to dogs with stable epileptic history under long-term antiepileptic therapy and may not extrapolate to healthy or other clinical populations. Further studies are required using larger cohorts with control variables to validate our observations.

## Conclusion

Here, we evaluated whether a single-day fecal sampling could reliably represent a three-day sampling protocol for assessing the dog gut microbiome. We observed stability of the gut bacterial community over three consecutive days, suggesting that one-day sampling is a valid alternative approach. These findings hold significant practical implications for both research and clinical applications, as they simplify the sampling process without compromising accuracy. By minimizing the time and cost tied to prolonged sampling designs, this approach can facilitate more efficient studies in canine gut microbiology, making single-day fecal sampling a good candidate for further exploration to validate these findings across diverse scenarios and to explore the broader applicability of this streamlined methodology in both veterinary sciences and other microbiome-related research fields. Future studies should incorporate broader health profiles and age groups to assess the consistency of these findings in diverse canine populations.

## Data Availability

The datasets presented in this study can be found in online repositories. The names of the repository/repositories and accession number(s) can be found in the article/Supplementary material.
