# Supplementary material for: Limited day-to-day variation in the canine gut microbiota: implications for microbiome studies
Source: Front Vet Sci. 2025 Jul 28;12:1632686. doi: 10.3389/fvets.2025.1632686 (PMC12337483; doi:10.3389/fvets.2025.1632686)
Supplement: Supplementary file 1 [file Table_1.docx]

**Supplementary Table 1: Detailed sample information**

| **Dog Codes** | **Age (years)*** | **Sex** | **Breed** | **Fecal Sample Day 1** | **Fecal Sample Day 2** | **Fecal Sample Day 3** |
| --- | --- | --- | --- | --- | --- | --- |
| 1 | 3 | FS | Golden Retriever | 1_1 | 1_2 | 1_3 |
| 2 | 4 | MC | Mixed breed | 2_1 | 2_2 | 2_3 |
| 3 | 6.5 | MC | Rhodesian Ridgeback | 3_1 | 3_2 | 3_3 |
| 4 | 2 | MC | Mixed breed | 4_1 | 4_2 | 4_3 |
| 5 | 4 | FS | Border Collie | 5_1 | 5_2 | 5_3 |
| 6 | 6.5 | MC | Border Collie | 6_1 | 6_2 | 6_3 |
| 7 | 5 | FI | Golden Retriever | 7_1 | 7_2 | 7_3 |
| 8 | 7 | FS | Jack Russell Terrier | 8_1 | 8_2 | 8_3 |
| 9 | 5 | FS | French Bulldog | 9_1 | 9_2 | 9_3 |
| 10 | 5 | MC | Border Collie | 10_1 | 10_2 | 10_3 |
| 11 | 2 | MC | Mixed breed | 11_1 | 11_2 | 11_3 |
| 12 | 6 | MC | Golden Retriever | 12_1 | 12_2 | 12_3 |

*Rounded to the nearest half year

Abbreviations: MC: male castrated; FS: female spayed; FI: female intact
